# Supplementary material for: Dynamic label-free analysis of SARS-CoV-2 infection reveals virus-induced subcellular remodeling
Source: Nat Commun. 2024 Jun 11;15:4996. doi: 10.1038/s41467-024-49260-7 (PMC11166935; doi:10.1038/s41467-024-49260-7)
Supplement: Supplementary file 1 — Supplementary information file [file 41467_2024_49260_MOESM1_ESM.pdf]

Dynamic label-free analysis of SARS-CoV-2 infection reveals virus-induced subcellular remodeling

Nell Saunders[1], Blandine Monel[1], Nadège Cayet[2], Lorenzo Archetti[3,†], Hugo Moreno[3,†], Alexandre Jeanne[3,†], Agathe Marguier[3], Julian Buchrieser[1], Timothy Wai[4], Olivier Schwartz[1,5]*, Mathieu Fréchin[3,]*.

[1]Virus & Immunity Unit, Institut Pasteur, Université Paris Cité, CNRS UMR 3569, Paris, France.
[2]Institut Pasteur, Université Paris Cité, Ultrastructural Bioimaging Unit, 75015 Paris, France
[3]Deep Quantitative Biology Department, Nanolive SA, Tolochenaz, Switzerland.
[4]Mitochondrial Biology Group, Institut Pasteur, Université Paris Cité, CNRS UMR 3691, Paris, France.
[5]Vaccine Research Institute, Creteil, France

[†]Contributed equally,
*Correspondence: Olivier.schwartz@pasteur.fr or Mathieu.frechin@nanolive.ch

Supplementary Information

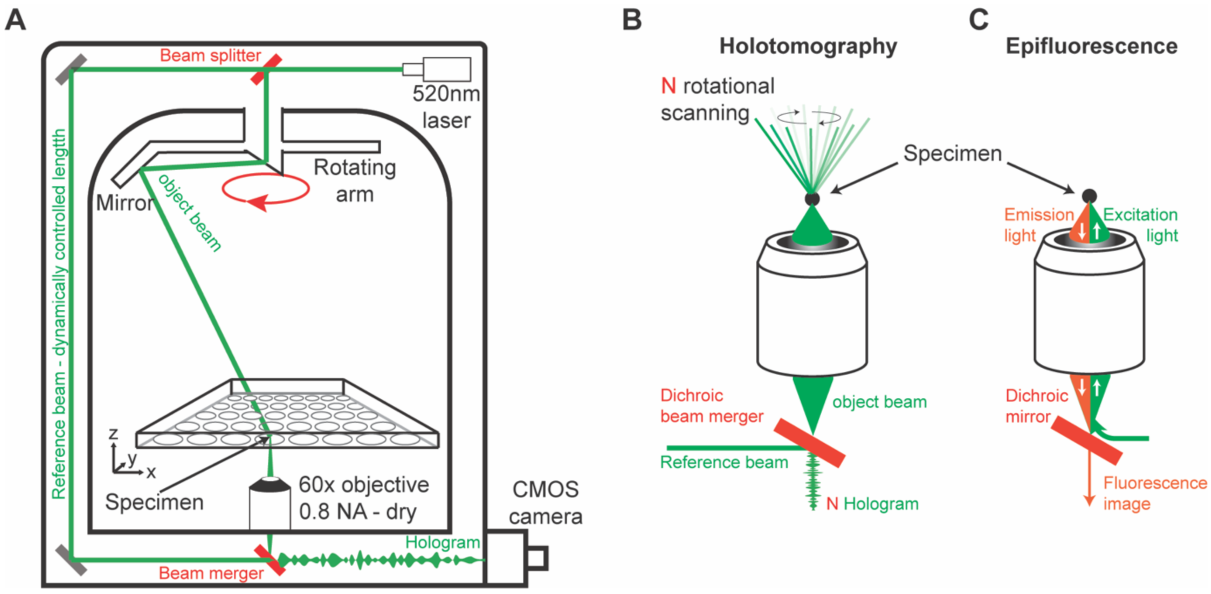

Supplementary figure 1 | Holotomographic microscope device schematics. (**A**) Overview of the microscope inner setting summarizing the holographic optical setup, the rotational scanning system, the adaptive optical path of the reference beam and the automated stage. (**B**) The rotation all over the specimen allows for the light to interact with the sample from different angles. The diffused light is collected by a 60x objective and brought to interference with the reference beam on a 50/50 dichroic beam merger, which creates as many holograms as there is illumination of the sample during the rotational scanning. (**C**). The device is equipped with an epifluorescence system.

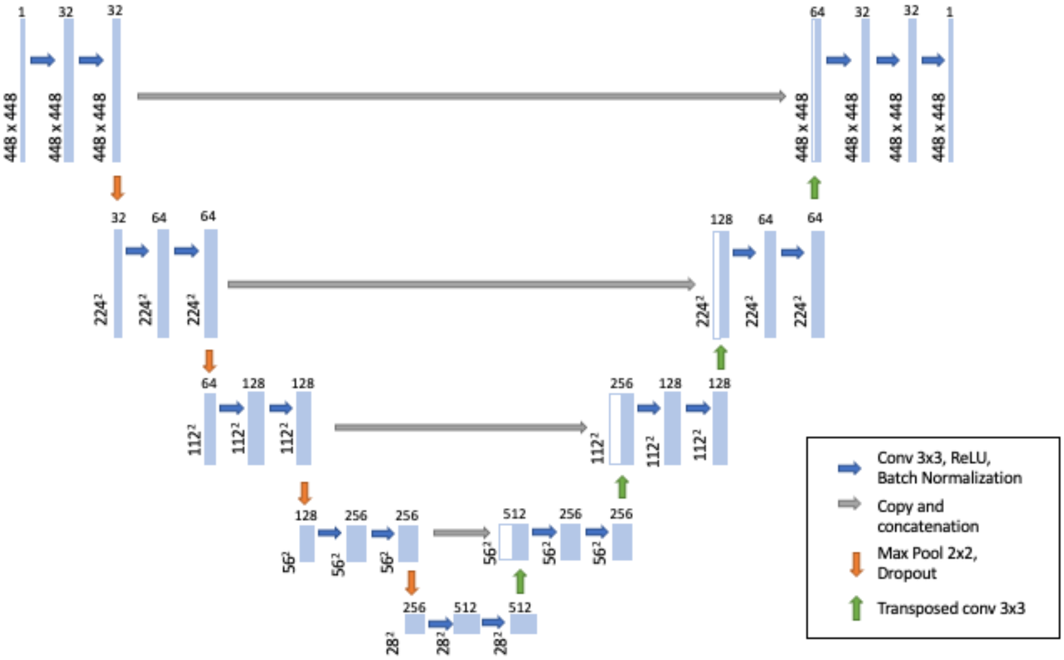

Supplementary figure 2 | Adapted UNET architecture used for cell, nuclei and nucleoli detection. Our deep learning segmentation model uses the well-established UNET architecture[34]. We modified it further by adding batch normalization as well as dropout steps that were key to our organelle detection performances. Batch normalization[36] reduces internal covariate shift via the fixing of the means and variances of layer inputs. It improves gradient flow and removes its dependence to parameters scale or initial values. The expected impact of batch normalization is quicker learning and less divergence. The dropout[37] step is also a regularization technique where each neuron of the network has a probability to be switched off (or dropped) at each training cycle. It aims at avoiding that few connections overtake the network's behavior, a process known as co-adaptation leading to overfitting.

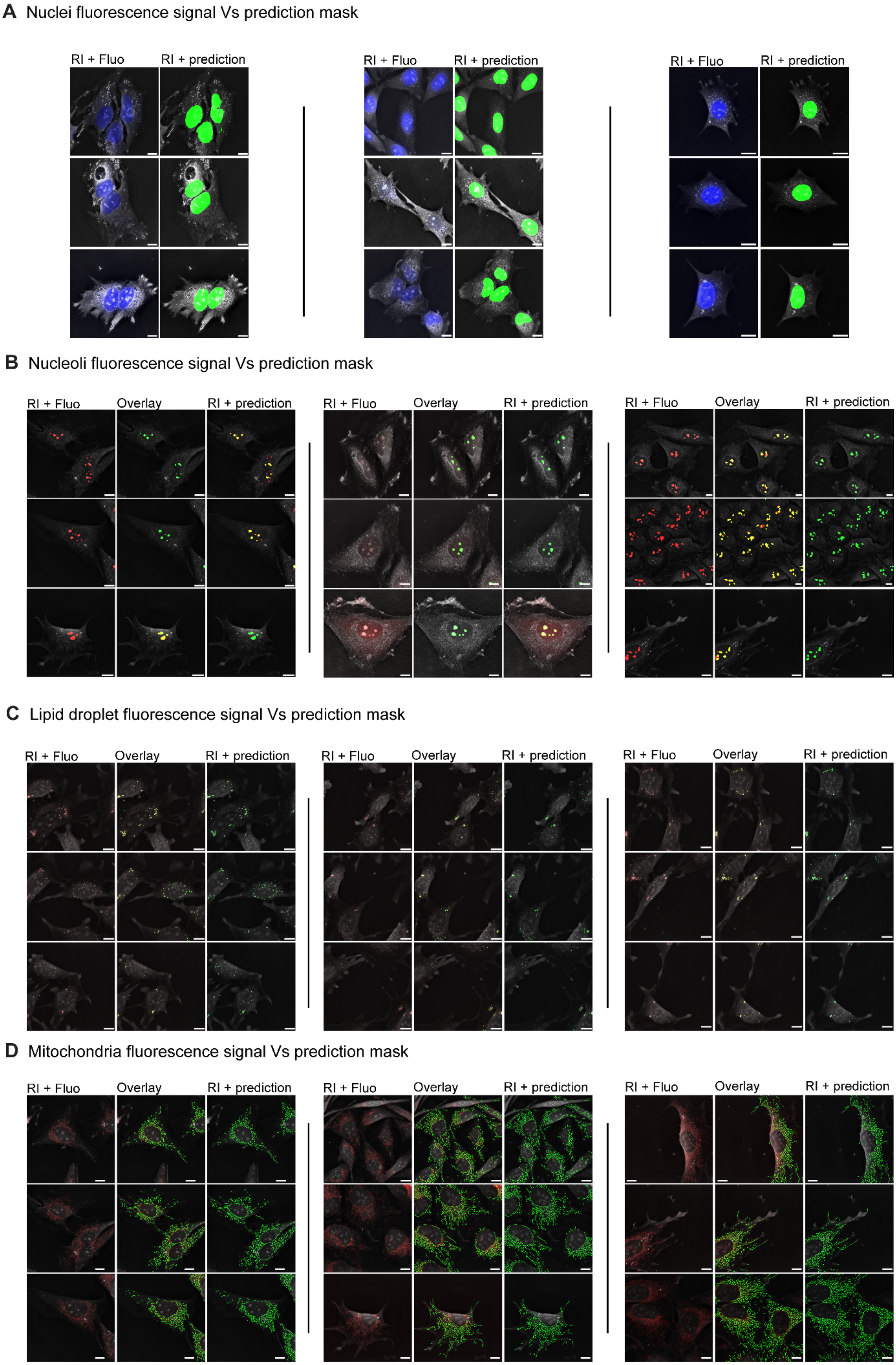
A Nuclei fluorescence signal Vs prediction mask
RI + Fluo
RI + prediction
B Nucleoli fluorescence signal Vs prediction mask
RI + Fluo
Overlay
RI + prediction
C Lipid droplet fluorescence signal Vs prediction mask
RI + Fluo
Overlay
RI + prediction
D Mitochondria fluorescence signal Vs prediction mask
RI + Fluo
Overlay
RI + prediction

Supplementary figure 3 | Comparison of organelle fluorescent signal and machine learning-based organelle prediction. RI-refractive index, Fluo-Fluorescence signal (**A**) Various forms of Nuclei, (**B**) nucleoli, (**C**) lipid droplets and (**D**) mitochondria, are detected efficiently in cells of various forms and confluency. For metrics quantifying the quality of our predictions see figure 2G. Scale bar, 10 µm.

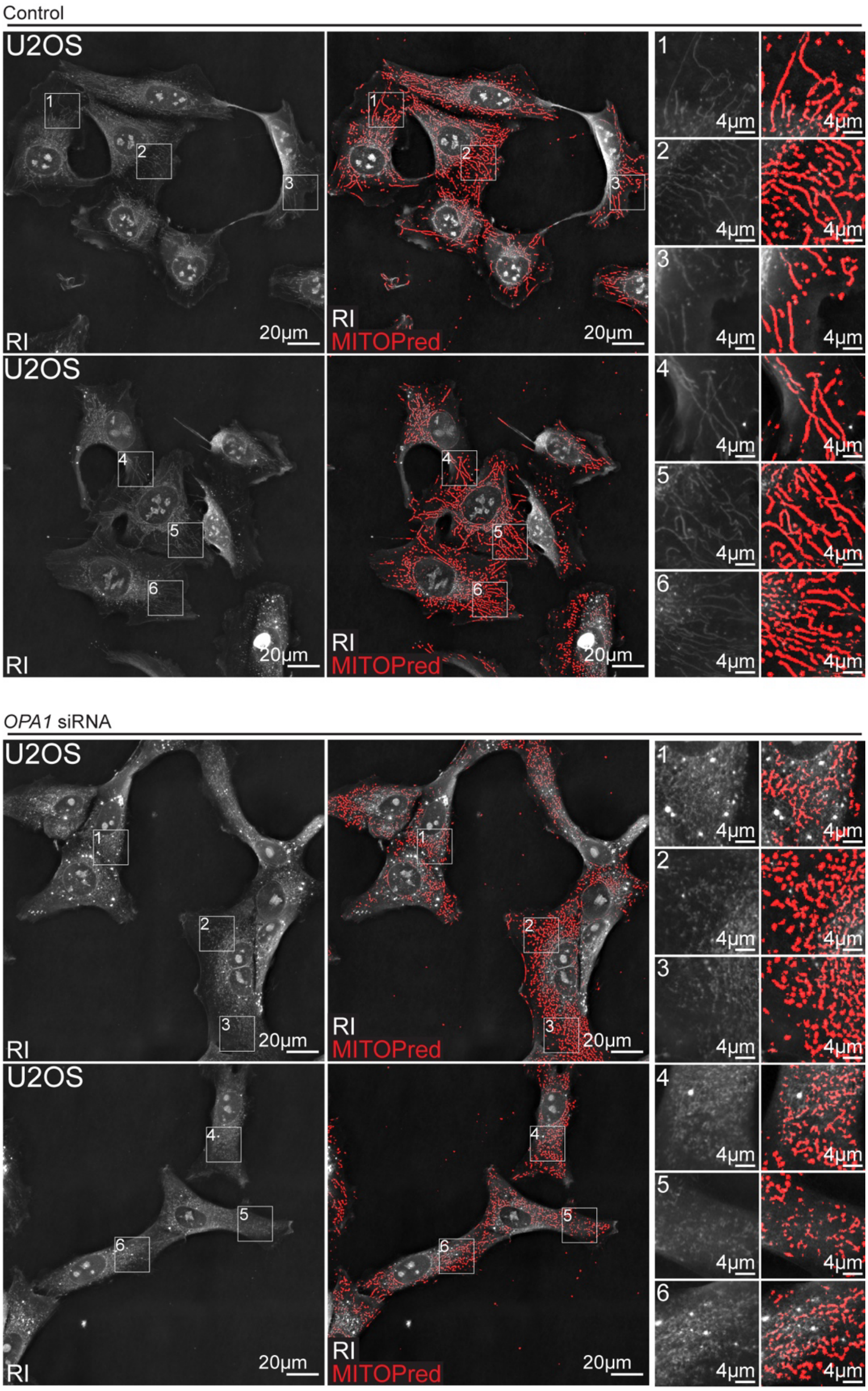

Supplementary figure 4 | Machine learning detects unperturbed or fragmented mitochondria after silencing of *OPA1*. Control U2OS cells display long, tubular, connected mitochondria, while U2OS cells experiencing *OPA1* silencing have punctuated, over-fragmented mitochondria. Zooms show that mitochondria are detected across a wide range of various morphologies. MITOPred-mitochondria predictions.

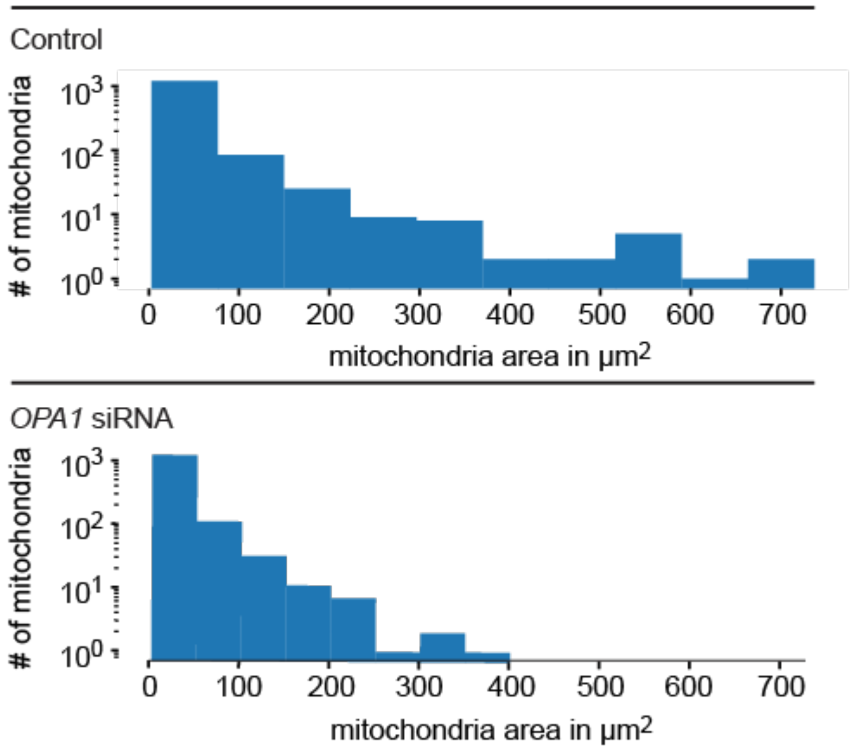

Supplementary figure 5 | The effect of *OPA1* silencing is quantified through automated mitochondria segmentation.

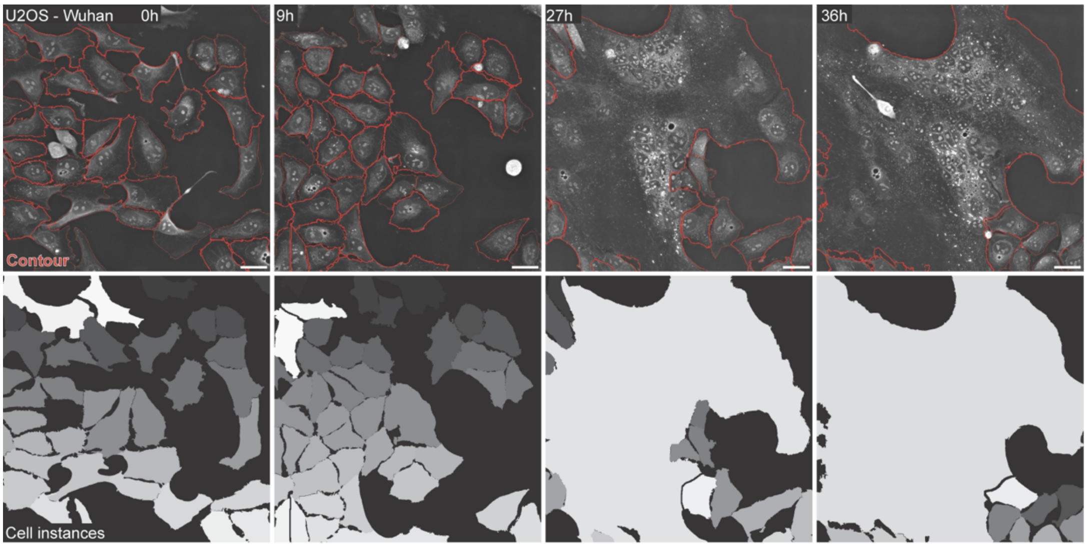

Supplementary figure 6 | Deep learning-based label-free cell segmentation detects single cells and syncytium with precision. At 0 and 9 hours, we can see that cells are all efficiently detected. At 27 and 36 hours, one can observe that the large syncytium is well segmented as

well as the co-existing single cells that did not fuse with the syncytium, validating our proper cell vs syncytium segmentation robustness. All our cell vs syncytium proper segmentations can be verified in our Supplementary Movies 1-19.

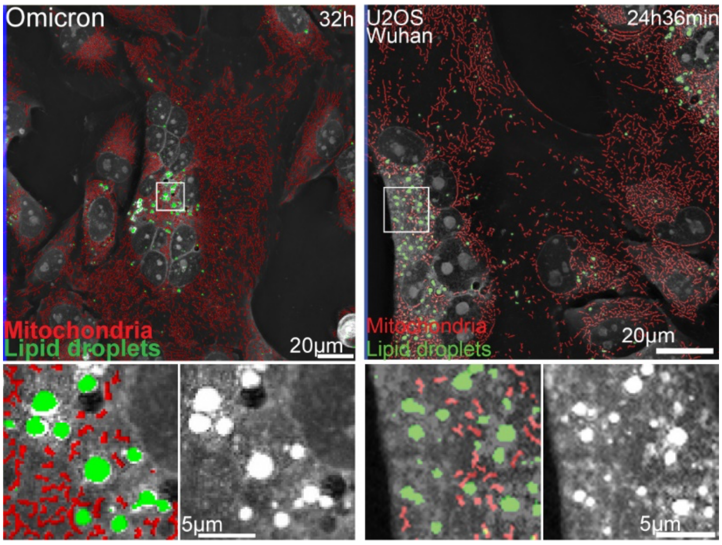

Supplementary figure 7 | Detection of SARS-CoV-2-induced lipid droplets in the perinuclear region of infectious syncytium.

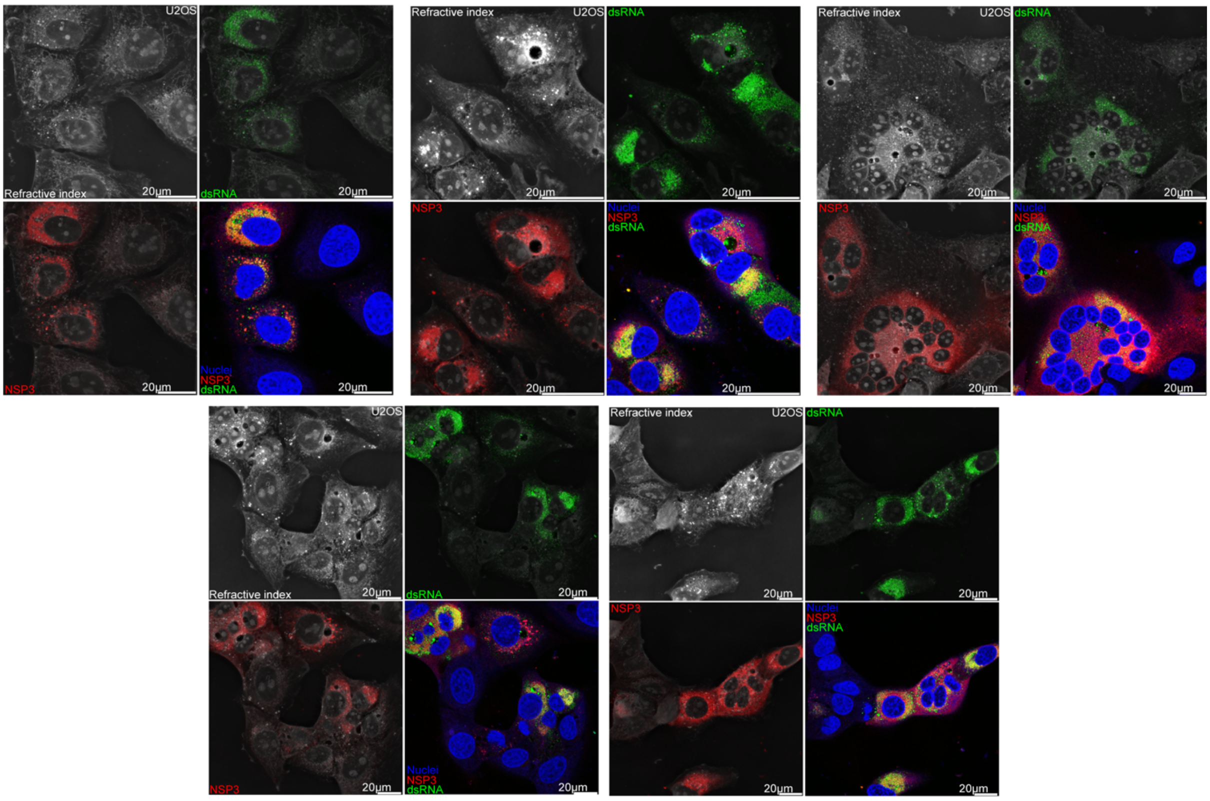

Supplementary figure 8 | Perinuclear lipid droplet accumulation is a marker of infection. Each of the five panels show that cells with large lipid droplets all show NSP3 and dsRNA immuno-fluorescent signal, while those with no lipid droplets do not display NSP3 and dsRNA immuno-fluorescent signal. See also figure 5.

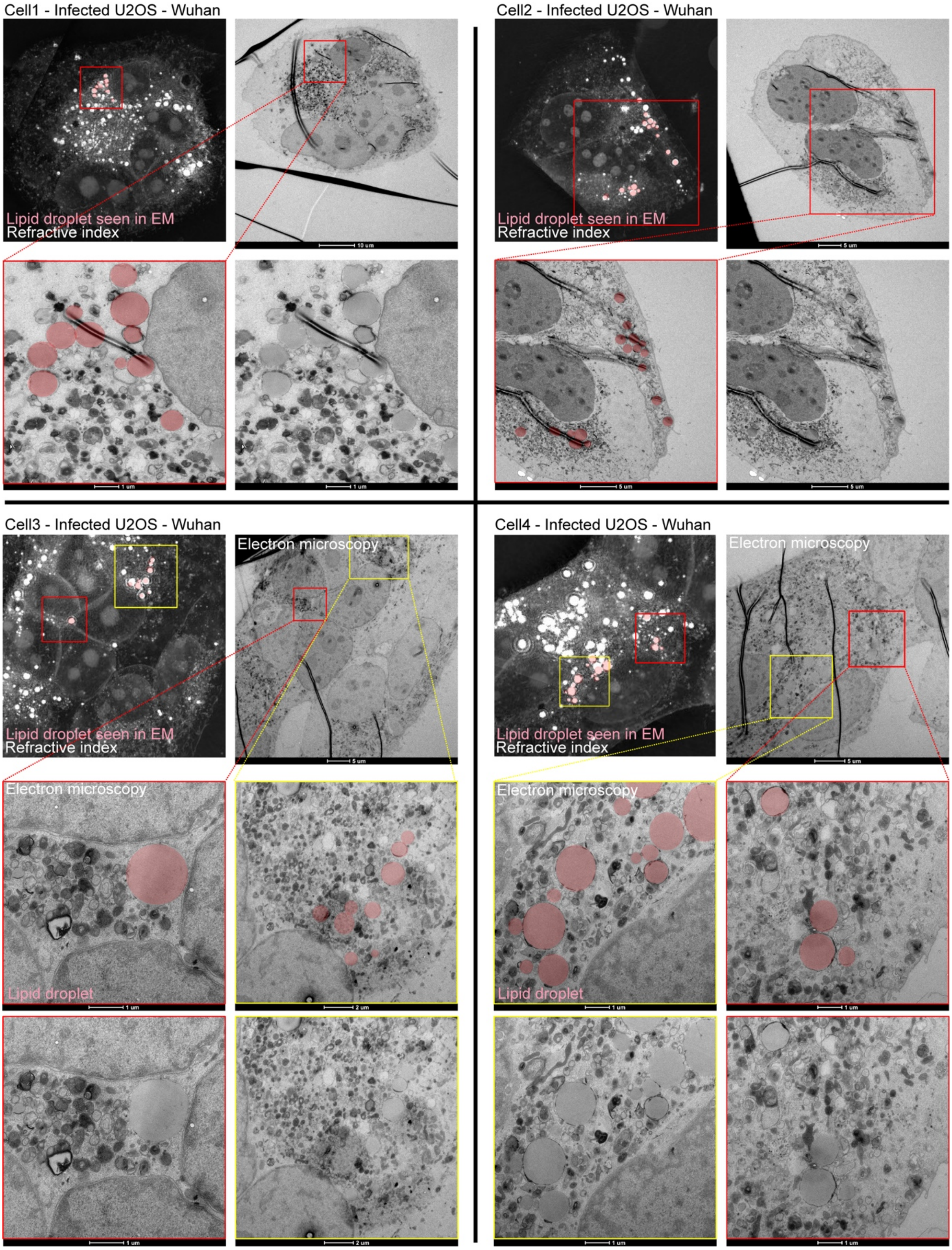

Supplementary figure 9 | Lipid droplets of infected cells are not surrounded by mitochondria. The four U2OS cells (cell 1, cell 2, cell 3, cell 4) and syncytium infected with the SARS-CoV-2 Wuhan strain and developing large lipid droplets do not show any mitochondria close to lipid droplets when observed by electron microscopy. See also figure 5E and 5F.

Edge	Covid score	Covid pVal	Syncytin 1 score	Syncytin 1 pVal
Cy>LD	260(15)	0	4(15)	0.9
Sy>LD	200(5)	0	2(5)	1
Cy>Sy	1(3)	0.9	33(3)	0

Supplementary table 1 | Pairwise statistical tests strengthen key organelle cross regulation (OCR) rewiring differences between infected cells and cells expressing Syncytin 1. Two-sided Chi-squared score and p-values reinforce the observation that cytosol and syncytium edges towards LD exists when cells are infected by SARS-CoV-2 but not when cells only express Syncytin 1.

Edge	freq.	score	DoF	pVal
Nx>Ni	0.84	2503	≤ 6	$<10^{-6}$
Nx>Cy	0.80	6700	≤ 6	$<10^{-6}$
Nx>LD	0.50	377	≤ 12	$<10^{-6}$
Nx>Mi	0.64	1326	≤ 6	$<10^{-6}$
Cy>Mi	0.32	481	≤ 9	$<10^{-6}$
Mi>Nx	0.36	747	≤ 6	$<10^{-6}$
Mi>LD	0.34	209	≤ 18	$<10^{-6}$
Mi>Cy	0.68	1041	≤ 9	$<10^{-6}$

Supplementary table 2 | Key metrics of unperturbed cell organelle cross regulation (OCR) network. Each edge of the OCR is described by its frequency over bootstrapping iterations, its mean two-sided chi-squared score, its maximum observed degree of freedom and p-Value.

Edge	freq.	score	DoF	pVal
Nx>Ni	0.81	1843	≤ 12	$<10^{-6}$
Nx>Cy	0.76	2786	≤ 9	$<10^{-6}$
Nx>Mi	0.60	1194	≤ 6	$<10^{-6}$
Cy>LD	0.67	260	≤ 15	$<10^{-6}$
Mi>Nx	0.40	764	≤ 6	$<10^{-6}$
Mi>Cy	0.68	1318	≤ 6	$<10^{-6}$
LD>Sy	0.75	644	≤ 5	$<10^{-6}$
Sy>LD	0.25	200	≤ 5	$<10^{-6}$

Supplementary table 3 | Key metrics of SARS-CoV-2-infected cell organelle cross regulation (OCR) network. Each edge of the OCR is described by its frequency over bootstrapping iterations, its mean two-sided chi-squared score, its maximum observed degree of freedom and p-Value.

Edge	freq.	score	DoF	pVal
Nx>Ni	0.91	456	≤9	$<10^{-6}$
Nx>Mi	0.33	571	≤6	$<10^{-6}$
Mi>Nx	0.67	1308	≤6	$<10^{-6}$
Mi>Cy	0.67	1647	≤6	$<10^{-6}$
Cy>Mi	0.33	812	≤6	$<10^{-6}$
Cy>Sy	0.70	33	≤3	$<10^{-6}$

Supplementary table 4 | Key metrics of Syncytin-1-expressing cell organelle cross regulation (OCR) network. Each edge of the OCR is described by its frequency over bootstrapping iterations, its mean two-sided chi-squared score, its maximum observed degree of freedom and p-Value.

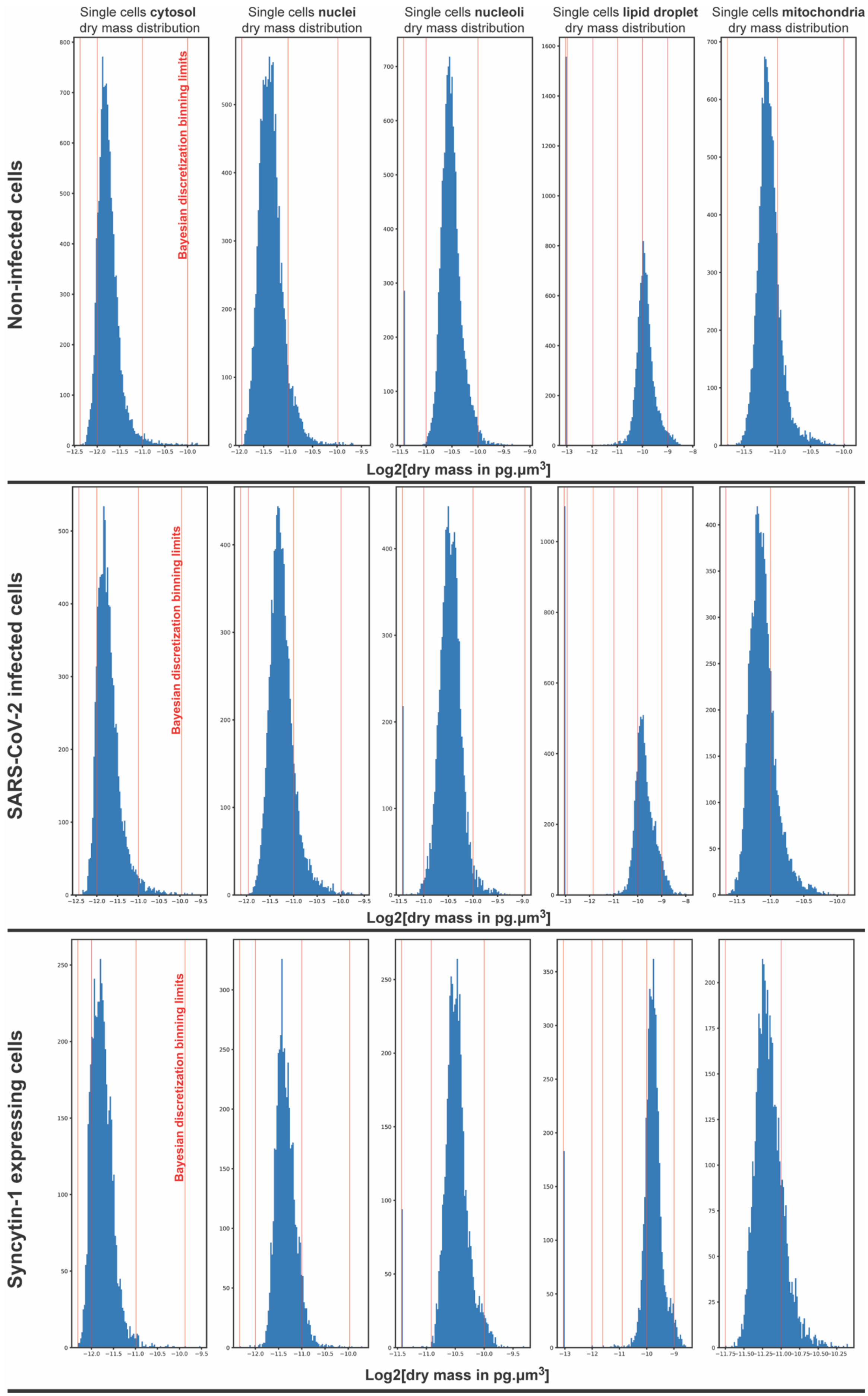
Single cells cytosol dry mass distribution
Single cells nuclei dry mass distribution
Single cells nucleoli dry mass distribution
Single cells lipid droplet dry mass distribution
Single cells mitochondria dry mass distribution
Non-infected cells
SARS-CoV-2 infected cells
Syncytin-1 expressing cells
Bayesian discretization binning limits
Log2[dry mass in pg.µm3]

Supplementary figure 10 | Distribution of single cells according to their respective dry mass located within the cytosol, nuclei, nucleoli, lipid droplets and mitochondria. Each line of five histograms represents single cells distributions (in blue) of a specific experimental condition (non-infected, infected, and syncytin-1-expressing cells) as well as their respective Bayesian binning represented as overlaying red lines.

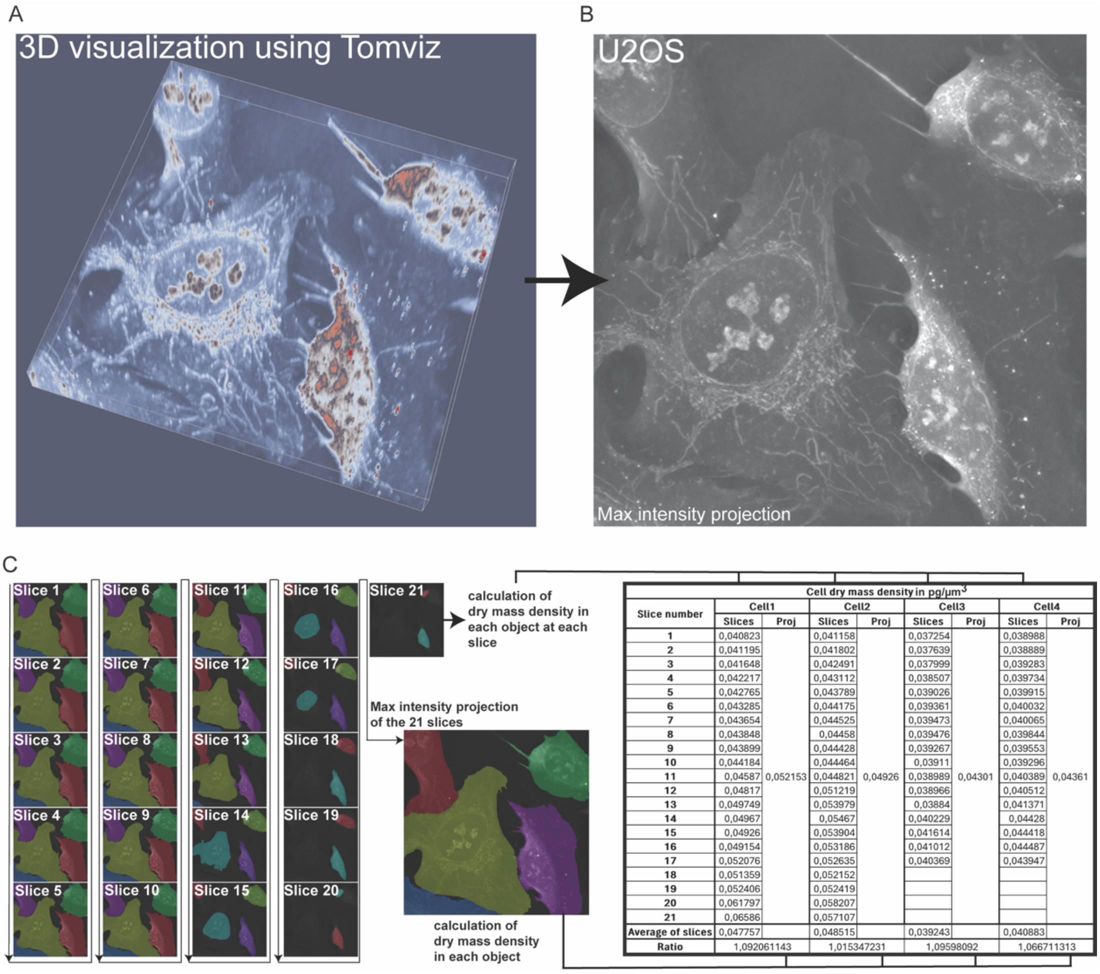

Slice number	Cell dry mass density in pg/µm³							
	Cell1		Cell2		Cell3		Cell4	
	Slices	Proj	Slices	Proj	Slices	Proj	Slices	Proj
1	0,040823		0,041158		0,037254		0,038988	
2	0,041195		0,041802		0,037639		0,038889	
3	0,041648		0,042491		0,037999		0,039283	
4	0,042217		0,043112		0,038507		0,039734	
5	0,042765		0,043789		0,039026		0,039915	
6	0,043285		0,044175		0,039361		0,040032	
7	0,043654		0,044525		0,039473		0,040065	
8	0,043848		0,04458		0,039476		0,039844	
9	0,043899		0,044428		0,039267		0,039553	
10	0,044184		0,044464		0,03911		0,039296	
11	0,04587	0,052153	0,044821	0,04926	0,038989	0,04301	0,040389	0,04361
12	0,04817		0,051219		0,038966		0,040512	
13	0,049749		0,053979		0,03884		0,041371	
14	0,04967		0,05467		0,040229		0,04428	
15	0,04926		0,053904		0,041614		0,044418	
16	0,049154		0,053186		0,041012		0,044487	
17	0,052076		0,052635		0,040369		0,043947	
18	0,051359		0,052152					
19	0,052406		0,052419					
20	0,061797		0,058207					
21	0,06586		0,057107					
Average of slices	0,047757		0,048515		0,039243		0,040883	
Ratio	1,092061143		1,015347231		1,09598092		1,066711313	

Supplementary figure 11 | Maximum intensity projection of holotomographic 3D volumes recapitulate volume-derived dry mass densities. (A) Holotomographic microscopy (HTM) returns 3D refractive index maps (B) are transformed into maximal intensity projection images. (C) The dry mass densities calculated from manual 3D cell segmentations, or automated 2D cell segmentations provide similar results, confirming that 2D maximal intensity projections recapitulate well the whole cell content.
