# Supplementary material for: Dynamic label-free analysis of SARS-CoV-2 infection reveals virus-induced subcellular remodeling
Source: Nat Commun. 2024 Jun 11;15:4996. doi: 10.1038/s41467-024-49260-7 (PMC11166935; doi:10.1038/s41467-024-49260-7)
Supplement: Supplementary file 24 — Description of Additional Supplementary Files [file 41467_2024_49260_MOESM24_ESM.pdf]

Description of Additional Supplementary Files

Supplementary Movie 1: Control uninfected U2OS-ACE2 cells. Seven representative movies of cells and organelles masks overlaid on refractive index signal.

Supplementary Movie 2: Control uninfected U2OS-ACE2 cells. Seven representative movies of cells and organelles masks overlaid on refractive index signal.

Supplementary Movie 3: Control uninfected U2OS-ACE2 cells. Seven representative movies of cells and organelles masks overlaid on refractive index signal.

Supplementary Movie 4: Control uninfected U2OS-ACE2 cells. Seven representative movies of cells and organelles masks overlaid on refractive index signal.

Supplementary Movie 5: Control uninfected U2OS-ACE2 cells. Seven representative movies of cells and organelles masks overlaid on refractive index signal.

Supplementary Movie 6: Control uninfected U2OS-ACE2 cells. Seven representative movies of cells and organelles masks overlaid on refractive index signal.

Supplementary Movie 7: Control uninfected U2OS-ACE2 cells. Seven representative movies of cells and organelles masks overlaid on refractive index signal.

Supplementary Movie 8: U2OS-ACE2 cells infected with SARS-CoV-2 Wuhan strain. Five representative movies of cells and organelles masks overlaid on refractive index signal.

Supplementary Movie 9: U2OS-ACE2 cells infected with SARS-CoV-2 Wuhan strain. Five representative movies of cells and organelles masks overlaid on refractive index signal.

Supplementary Movie 10: U2OS-ACE2 cells infected with SARS-CoV-2 Wuhan strain. Five representative movies of cells and organelles masks overlaid on refractive index signal.

Supplementary Movie 11: U2OS-ACE2 cells infected with SARS-CoV-2 Wuhan strain. Five representative movies of cells and organelles masks overlaid on refractive index signal.

Supplementary Movie 12: U2OS-ACE2 cells infected with SARS-CoV-2 Wuhan strain. Five representative movies of cells and organelles masks overlaid on refractive index signal.

Supplementary Movie 13: U2OS-ACE2 cells infected with SARS-CoV-2 Omicron BA.1. Four representative movies of cells and organelles masks overlaid on refractive index signal.

Supplementary Movie 14: U2OS-ACE2 cells infected with SARS-CoV-2 Omicron BA.1. Four representative movies of cells and organelles masks overlaid on refractive index signal.

Supplementary Movie 15: U2OS-ACE2 cells infected with SARS-CoV-2 Omicron BA.1. Four representative movies of cells and organelles masks overlaid on refractive index signal.

Supplementary Movie 16: U2OS-ACE2 cells infected with SARS-CoV-2 Omicron BA.1. Four representative movies of cells and organelles masks overlaid on refractive index signal.

Supplementary Movie 17: U2OS-ACE2 cells forming syncytia upon expression of Syncytin-1. Three representative movies of cells and organelles masks overlaid on refractive index signal.

Supplementary Movie 18 U2OS-ACE2 cells forming syncytia upon expression of Syncytin-1. Three representative movies of cells and organelles masks overlaid on refractive index signal.

Supplementary Movie 19: U2OS-ACE2 cells forming syncytia upon expression of Syncytin-1. Three representative movies of cells and organelles masks overlaid on refractive index signal.

Supplementary Movie 20: Control uninfected U2OS-ACE2 cells acquired at high temporal resolution of one image every two minutes.
